# Impact of the COVID-19 pandemic on the willingness to sacrifice for the environment: The Austrian case

**DOI:** 10.1007/s11614-021-00464-x

**Published:** 2021-11-28

**Authors:** Beate Klösch, Rebecca Wardana, Markus Hadler

**Affiliations:** Institute of Sociology, University of Graz, Universitätsstraße 15, 8010 Graz, Austria

**Keywords:** Environment, Crisis, Attitudes, Environmentally significant behavior, Willingness, Umwelt, Krise, Einstellungen, Umweltverhalten, Werte

## Abstract

Previous analyses of environmentally conscious intentions showed that the willingness to sacrifice for the environment decreased during the COVID-19 crisis in Austria. There is a large body of empirical research and theoretical models dealing with the explanation of environmental behavior, but these explanations have always been considered in the context of a pandemic-free society. The aim of this research note is therefore to consider the willingness to sacrifice in a crisis period. The data used for the analyses is the Austrian part of the international ‘Values in Crisis’ survey. For this purpose, more than 2000 individuals were surveyed online about their values, social orientations and their current life situation during the first COVID-19 wave (May 2020). Blockwise regression models are used to examine the influence of crisis perceptions, environmental attitudes and values on the willingness to sacrifice for the environment. The analyses show a relatively strong influence of environmental attitudes and values, but also additional effects of concerns about the COVID-19 crisis and especially its economic impact.

## Abstract (de)

Erste Analysen zu Umwelteinstellungen und Umweltverhalten in Österreich zeigten, dass die Bereitschaft, umweltbewusst zu handeln, im Verlauf der COVID-19-Krise gesunken ist. Es gibt zahlreiche empirische Forschungen und theoretische Modelle, die sich mit der Erklärung von Umweltverhalten beschäftigen, jedoch wurden diese Erklärungen stets im Kontext einer pandemiefreien Gesellschaft betrachtet. Ziel dieses Beitrags ist es daher, die Verhaltensintention für umweltfreundliches Handeln in einem krisenzeitlichen Kontext zu betrachten. Der für die Berechnungen verwendete Datensatz ist der österreichische Teil der internationalen „Values in Crisis“-Erhebung. Dazu wurden über 2000 Personen zum Zeitpunkt der ersten COVID-19-Welle (Mai 2020) zu ihren Werten, sozialen Orientierungen und ihrer aktuellen Lebenssituation in der Krise online befragt. Mittels Regressionsmodellen werden die Wahrnehmung der Krise sowie der Einfluss unterschiedlicher Umwelteinstellungen und Werte auf die Verhaltensintention für umweltbewusstes Handeln untersucht. Die Analysen zeigen einen starken Einfluss der Umwelteinstellungen und Werte, aber auch zusätzliche Einflüsse der individuellen Besorgnis über COVID-19, vor allem über ökonomische Auswirkungen.

## Introduction

Climate change, the climate crisis, and related movements were the main topics in the media throughout 2019 culminating in the election of Greta Thunberg as *Time Magazine*’s person of the year. The advent of the COVID-19 crisis, however, brought a sudden end to this attention and shifted the public interest to the pandemic. A descriptive analysis of Austrian survey data showed that the individual concern regarding the environment declined (Wardana et al. 2021). Yet, it is not clear if the COVID-19 crisis also altered the underlying relationship between environmental attitudes, concerns, and the willingness to sacrifice for the environment among Austrians.

An effect of the pandemic on the willingness to sacrifice can be expected given that the COVID-19 crisis is associated with severe health concerns and increased economic hardship—the unemployment rate in Austria, for example, jumped from 8.1 to 12.3% from February to March 2020 (Kremer and Wanek-Zajic 2020). Both factors, concerns about health and financial hardship are associated with environmental attitudes and behavioral intentions, as we will point out in the theory section, and are predicted to lower an individual’s willingness to make a sacrifice.

Our research note is organized as follows. The subsequent theory section situates our dependent variable at the border between environmental concerns and behaviors and then presents literature on the association between economic hardship, health concerns, and environmental concerns. The methods section introduces our data—the Austrian sample of the international “Values in Crisis” (VIC) survey project (Aschauer et al. 2020)—and our analysis strategies.

The results section shows that concerns surrounding COVID-19 do have effects on the willingness to sacrifice for the environment, but also that the underlying relationship between environmental attitudes and the willingness to make sacrifices remains strong. We discover differentiated effects of COVID-19 perceptions—while the impact of economic concerns remains significant when controlled for environmental concerns and values, health concerns seem to overlap with the benevolence and universalism dimension of Schwartz’s theory. Future research we conclude, needs to check whether or not these effects are lasting.

## Theoretical background

We focus on the willingness to make sacrifices for the environment. Considering the various measures of environmental attitudes, behaviors, and concerns, Mayerl and Best (2019) aimed to situate this variable in the existing literature. They concluded that it reflects the behavioral dimension of the tripartite classification of Maloney’s and Ward’s (1973) ecology scale and that it is also part of Dunlap’s and Jones’ (2002) environmental concern. Further, it can be seen as a behavioral intention variable that fits well with Ajzen’s and Fishbein’s (1980) attitude-behavior model. Finally, following Stern’s (2000) classification of environmentally significant behavior, our interest lies in nonactivist behaviors in the public sphere.

Considering empirical research that focuses on the predictors of environmental behavior at the more general level shows that values and attitudes regarding the environment are mentioned frequently (see, among others, Schwartz’s Theory of Basic Values 1992, 2012; Inglehart’s postmaterialism theory 1977, 1981, 2008; Diekmann and Preisendörfer 1992; Stern 2005; Schultz et al. 2005). According to Schwartz, environmental concerns are stronger among individuals with high self-transcendence, which is divided into benevolence and universalism. The same applies to individuals with strong post-materialist values (Inglehart). People with high scores in benevolence try to preserve and enhance the welfare of their in-group members, whereas individuals with high levels of universalism aim to understand, appreciate, tolerate and protect the welfare of all people and nature, which often comes along with a strong commitment towards social justice and environmental sustainability (see Schwartz 2012). Alongside these values and attitudes, various sociodemographic variables such as age, gender, education, income as well as political orientation have been found to be influential on environmental behavior (see Kollmuss and Agyeman 2002; Stern 2005; Hadler and Haller 2013; Huddart Kennedy et al. 2015; Hadler 2016; among others). Most studies find positive effects of education, income, political-left orientation, gender (female) and negative effects of age on the willingness to make sacrifices for the environment.

It is unclear how the impact of the COVID-19 crisis affects these predictors of environmental attitudes and behaviors. However, research on the effect of crises on individuals have shown that economic crises such as the recession of 2008 have a negative impact on the willingness to pay for climate change mitigation (Ivlevs 2019). High unemployment rates in particular have a significant negative effect on individuals prioritizing environmental protection (Scruggs and Benegal 2012; Kenny 2020; Duijndam and van Beukering 2021). Research has also pointed to several links between environmental attitudes and health concerns (see for example Lichtenberg and Zimmerman 1999; McCright and Xiao 2014) as well as to the competing effects of health and environmental considerations in behavior decisions (Karp 1996; Swenson and Wells 2018). Considering these findings on the effects of crises, we propose the following hypothesis:

### Hypothesis

The greater a person’s concerns that they or someone close will experience economic hardship due to the COVID-19 crisis or the greater the worry that they or someone close will contract COVID-19, the lower the willingness to make sacrifices for the environment.

## Data and methods

We use the Austrian sample of the international “Values in Crisis” (VIC) project (Aschauer et al. 2020), which was collected during the first lockdown in May 2020. The VIC-project is an international cooperation initiated by the World Values Survey (WVS) group. The questionnaire includes a standard set of WVS questions, COVID-19 related items, and additional national variables. The Austrian research group added, among other questions, items on environmental attitudes and concerns, which are used in our analysis.

The data was collected online and the sample was drawn from an online panel with 128,500 participants, due to restrictions for other fieldwork methods during the lockdown. The same panel has been used for other benchmark studies such as the Austrian Corona Panel Project and the Austrian National Election Study (see Kittel et al. 2021). The selection of the respondents is based on a stratified quota sampling strategy considering gender, age, region, and education (Aschauer et al. 2020).

Our final sample comprises 2018 respondents. It includes almost equal numbers of men (49.2%) and women (50.8%). The average age is 46 and slightly above the Austrian average of 42.8 years. The educational groups roughly correspond to the Austrian distribution, but respondents with a high-school (Matura) degree (19.7%) as well as an apprenticeship degree (39%) are slightly overrepresented, while individuals with a university degree are slightly underrepresented (10.8%). The average monthly net household income of the sample is around 2860 Euros and above the Austrian average of 2301 Euros (see Statistik Austria 2020a, b, c).

**Fig. 1:**
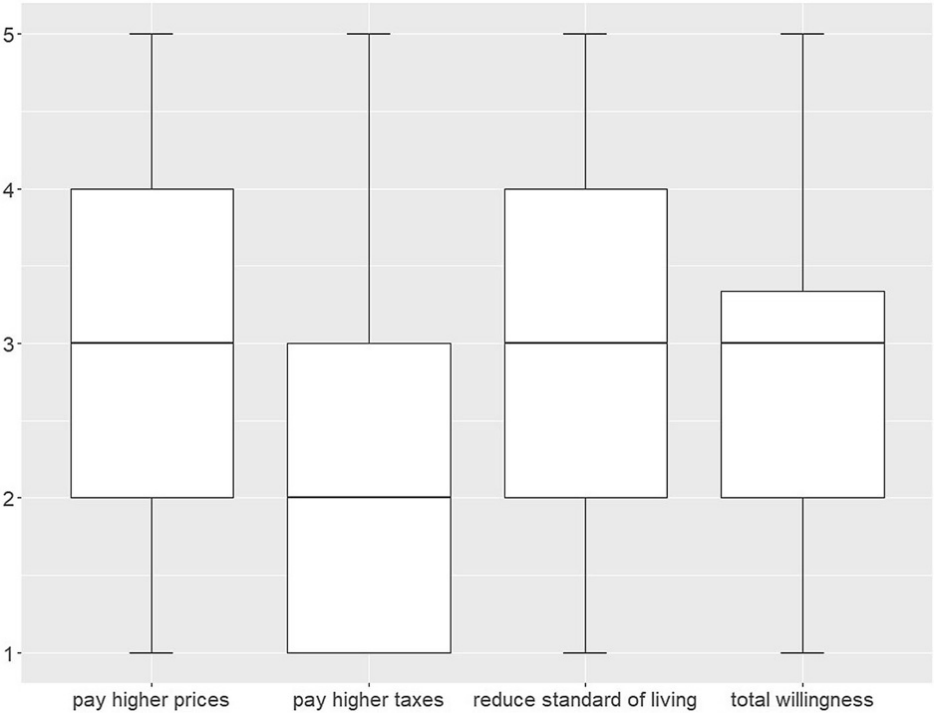
Distribution of main variables (willingness to …)^a^. (^a^Low value on the different variables indicates low willingness to sacrifice for the environment; the total willingness represents the mean score of all three previous variables)

To measure a respondent’s willingness to make sacrifices for the environment, two items were used that asked about the acceptance of financial measures and one item on the acceptance of restrictions in living standards. The wordings are “How willing would you be to … a) pay much higher prices; b) pay higher taxes, and c) accept cuts in your standard of living … in order to protect the environment”. The items used a 5-point Likert scale ranging from “very willing” to “very unwilling”. We combined these items to a summative index (Cronbach’s Alpha = 0.789) of an individual’s willingness to sacrifice for the environment (see also Table 1 and Fig. 1).

**Table 1:** Overview of variables and descriptive statistics

		Mean (Std. Dev) or %
**Main Variable**		
*Willingness*	*To what extent would you personally find it acceptable for you to …* (1 = very unacceptable; 5 = very acceptable)	
	… pay much higher prices to protect the environment	2.82 (1.16)
	… pay much higher taxes to protect the environment	2.43 (1.14)
	… cut back on your standard of living in order to protect the environment	3.20 (1.14)
	*Index (mean score of all previous variables)*	2.82 (0.97)
**Independent variables**		
*Socio-demographic variables*	*Female*	50.8%
	*Age*	46 (18)
	*Education:*	
	Compulsory school	17.3%
	Apprenticeship	39%
	Intermediate vocational school	13.2%
	High school degree	19.7%
	University	10.8%
	*Net household income*	2858 €
	*Political orientation (1* *=* *left; 10* *=* *right)*	5.37 (1.99)
*Attitudes towards the COVID‑19 crisis*	How afraid are you that you yourself or people close to you will get sick with the COVID-19 virus and suffer a severe course of illness? (1 = low, 5 = high)	2.51 (1.05)
	How afraid are you that you yourself or people close to you will suffer from an economic hardship after the COVID-19 crisis? (1 = low, 5 = high)	2.80 (1.10)
*Values*	*Postmaterialism (1* *=* *materialism, 2* *=* *postmaterialism)* People sometimes talk about what the aims of this country should be for the next few years. On this card are listed some of the goals which different people would give top priority. Would you please say which one of these you, yourself, consider the most important? And which would be the next most important?	1.55 (0.5)
	1) Maintaining order in the nation	
	2) Giving people more say in important government decisions	
	3) Fighting rising prices	
	4) Protecting freedom of speech	
	*Universalism (1* *=* *low, 6* *=* *high)*	4.51 (0.92)
	Now you will see descriptions of some persons. Please look at each description and indicate how much each person is or is not like you	
	They believe it is important that all people in the world should be treated equally. They believe that everyone should have equal opportunities in life	
	It is important for them to listen to people who are different from them. Even if they disagree with others, they still want to understand them	
	They firmly believe that people should take care of nature. Environmental protection is important to them	
	*Benevolence (1* *=* *low, 6* *=* *high)*	4.79 (0.93)
	Now you will see descriptions of some persons. Please look at each description and indicate how much each person is or is not like you	
	It is very important to them to help the people around them. They want to take care of their well-being	
	It is important for them to be loyal to their friends. They want to stand up for people who are close to them	
*Environmental attitudes*	*Environmental concern (1* *=* *no worries, 5* *=* *very worried)*	3.60 (1.09)
	*Opinion on climate change:* There has been a lot of discussion recently about global climate and the view that it has changed over the last few decades. Which of the following statements do you think comes closest?	
	The global climate has not changed	3.3%
	The global climate has changed primarily through natural processes	11.3%
	The global climate has changed in roughly equal parts through natural processes and through human activity	33.0%
	The global climate has changed mainly due to human activity	52.3%
	I can’t tell	5.2%

A number of sociodemographic variables which are considered relevant in the context of environmental attitudes and behavior were used as independent variables (see for example Hadler and Wohlkönig 2012). In addition to these sociodemographic variables, values and attitudes concerning the environment were also included. Regarding environmental values we used items for postmaterialism (Inglehart) as well as universalism and benevolence (Schwartz). Environmental concern and attitudes towards climate change were also considered. Finally, two items were selected to illustrate personal concerns regarding COVID-19: a) how much one is afraid of contracting COVID-19; and b) how much one is afraid of the economic consequences of the COVID-19 crisis. Table 1 provides a summary of all variables used in our analyses.

The results section starts with a descriptive overview of our dependent variables. Subsequently, a blockwise regression model is used to test our hypothesis. A total of four models are presented that include different sets of variables (see Table 2). Using this approach of gradually adding different subject blocks, we can observe how the influence of previously relevant factors changes due to the added variables. Thus, we are able to determine whether the crisis dimension, represented by fear of COVID-19 disease as well as fear of economic hardship, changes the influence of previously relevant predictors of the willingness. For more information on the estimation approach of blockwise regression models see Backhaus et al. (2018).

**Table 2:** Listing of the blockwise regression models

Blockwise regression	Dimensions and variables used
Model 1	Basic model, includes only socio-demographic variables
Model 2	Model 1 + crisis level (fear of COVID-19 infection, fear of economic hardship due to COVID-19)
Model 3	Model 1 + value level (postmaterialism according to Inglehart, universalism and benevolence according to Schwartz) + attitude level (environmental concern, attitude towards climate change)
Model 4	Final model, includes all variables

## Results

Fig. 1 describes the distribution of the main variables. As already mentioned, the willingness of the respondents consists of a total of three variables, which represent the willingness to accept financial restrictions in form of higher prices, taxes and limitations in their standard of living for the benefit of the environment. The distributions of the individual variables show that respondents are generally more willing to accept an increase in prices and a reduction in their standard of living. Slightly more than 50% of the respondents are not willing to accept an increase in taxes in favor of the environment. Calculating the mean value of these three variables shows that the willingness to sacrifice for the environment is on average 2.8, which indicates a medium willingness. The boxplot of “total willingness” shows that in the third quartile, the distribution of those who tend to show a higher willingness is no longer so widely spread, compared to the first quartile, where there are people who tend to have a lower willingness.

After considering the distribution of our main variables, we now turn towards testing our hypothesis using the total willingness of a person as the main variable in our models.^1^ Table 3 presents the results of the four models, which were described in Table 2. Across the four models, an increase in the explained variance from 7% to almost 24% can be observed. This shows that the willingness can be explained much better on the basis of the added variables and that meaningful factors were selected for the analysis.

**Table 3:** Blockwise regression analyses of environmental willingness^a^

Independent variable	Willingness to sacrifice for the environment^b^ (1 = low, 5 = high)			
	Model 1	Model 2^c^	Model 3	Model 4
	Beta Coefficients	Beta Coefficients	Beta Coefficients	Beta Coefficients
*Intercept (unstandardized)*	3.437***	2.803***	1.922***	1.412***
*Sex/Gender (ref.: male)*	0.042	0.026	0.004	−0.009
*Age*	−0.023	−0.027	−0.048**	−0.053*
*Income*	0.058**	0.052*	0.070**	0.061**
*Education (ref.: university)*				
Compulsory school	−0.109**	−0.109**	−0.085**	−0.084**
Apprenticeship	−0.187***	−0.188***	−0.134***	−0.134***
Intermediate vocational school	−0.120***	−0.121***	−0.086**	−0.087**
High school degree	−0.042	−0.047	−0.023	−0.028
*Political Orientation (1* *=* *left, 10* *=* *right)*	−0.206***	−0.191***	−0.066**	−0.054*
*Fear of getting sick (0* *=* *no anxiety)*	–	0.312**	–	0.181
*Fear of getting sick*^*2*^	–	−0.206*	–	−0.098
*Fear of economic hardship (0* *=* *no anxiety)*	–	0.259*	–	0.250*
*Fear of economic hardship*^*2*^	–	−0.333**	–	−0.342**
*Inglehart’s Postmaterialism (ref: materialism)*	–	–	0.004	0.015
*Schwartz’s Universalism*	–	–	0.282***	0.286***
*Schwartz’s Benevolence*	–	–	−0.107***	−0.098***
*Environmental concern (0* *=* *no worries)*	–	–	0.202***	0.194***
*Opinion on climate change (ref.: man-made change* *=* *0)*				
Natural change	–	–	−0.131***	−0.120***
Both natural and man-made	–	–	−0.091***	−0.087***
No change	–	–	−0.032	−0.023
Can’t tell	–	–	−0.076***	−0.077***
*Explained variance (R*^*2*^* corr.)*	0.071***	0.093***	0.221***	0.237***
*Change in R*^*2*^	–	0.023	0.128	0.016

*p < 0.05, **p < 0.01, ***p < 0.001

^a^The initial calculations also included the area of residence in terms of city, suburb and countryside, the place of residence by state, the question about children and, in connection with values, the question about second-generation migration background. In all four models, these variables show no influence on the willingness to sacrifice for the environment, which is why they are no longer included in the final models

^b^Calculations were also performed where financial constraints and reductions in the standard of living were considered as separate dependent variables. However, the effects were very similar, which is why only the results for the combined index are presented here for the sake of brevity

^c^Calculations were also made with linear crisis variables: fear of getting sick (beta = 0.123***), fear of economic hardship (beta = −0.074**)

In the first model, the influence of relevant sociodemographic variables on the willingness to sacrifice for the environment is examined. These variables explain a total of 7.1% of the variance. Significant influences are found for income, education, and political attitude, with the latter having the strongest negative influence in the model (beta = −0.206). The further to the left the respondents consider themselves politically, the more willingness to sacrifice they show. The second strongest influence is exerted by the educational variables. Individuals with a lower degree (compulsory school, apprenticeship, or intermediate vocational school) show a lower willingness to sacrifice in comparison to respondents with a university degree. In addition, there is also a small positive influence of income, which means that individuals with a higher net household income show a higher willingness to sacrifice for the environment. These effects, which are shown in the first model, correspond to findings from previous empirical research.

In the second model, the two items on the perception of the COVID-19 crisis as well as their squared terms are added.^2^ The crisis variables seem to have significant influence on the willingness of the respondents to sacrifice for the environment, although the increase in explained variance is only minor (0.023). The effects of the beta coefficients of the crisis variables seem strong at this point of the analysis. Deriving from previous literature, we suspect a strong influence of environmental values and attitudes on the willingness and expect some change in the crisis effects, which we will come back to in the discussion of our final model.

In the third model, we wanted to take a closer look at the effects of environmental values and attitudes on the willingness to sacrifice for the environment. Therefore, Inglehart’s postmaterialism, elements of Schwartz’s Value-Theory (universalism and benevolence), environmental concern and a question about the origin of climate change are added. While Inglehart’s postmaterialism has no effect on the willingness to sacrifice, Schwartz’s items in universalism and benevolence have a significant effect. Universalism has a strong positive effect (beta = 0.282) while benevolence has a negative effect (beta = −0.107). The increase of the explained variance from 7.1 to 22.1% shows that these items have a high explanatory power. There is little change in the effects of the sociodemographic variables compared to the previous model, and the effects of education and political orientation become weaker.

The final model includes all of the previously mentioned variables. The explained variance increases to almost 24%. Comparing all value and attitude variables, universalism has the strongest influence within the final model (beta = 0.286), followed by environmental concern (beta = 0.194). Interestingly, the final model also shows a change of influence within the crisis variables. While in model 2 both crisis variables were significant predictors of the willingness to sacrifice, this changes in the final model when all variables are added. The fear of getting sick is not significant anymore while the fear of economic hardship remains significant and one of the strongest predictors in the final model. All effects of the sociodemographic variables (except gender) stay significant but have very low influence. Generally, it should be noted that the effects of the sociodemographic variables decrease slightly when adding variables related to values and attitudes.

## Discussion and conclusions

Our contribution started from the observation that the COVID-19 crisis lowered the public awareness of the climate crisis (Wardana et al. 2021) which resulted in the research question of whether or not the underlying relationship between environmental values and attitudes and the willingness to make sacrifices for the environment is affected. We differentiated between economic and health effects of the COVID-19 crisis and posited the following hypothesis: The greater a person’s concerns that they or someone close will experience economic hardship due to the COVID-19 crisis or that they or someone close will contract COVID-19, the lower the willingness to make sacrifices for the environment.

The analysis of public opinion data collected in Austria after the peak of the first COVID-19 wave in May 2020 shows that the effects of values, environmental attitudes and concerns on the willingness to sacrifice for the environment are still very strong and exceed the effects of socio-demographics and concerns about COVID-19. Yet, our findings indicate an additional influence of the worry about negative effects from the COVID-19 crisis, whereby the effects of economic worries are stronger than those of health aspects.

**Fig. 2:**
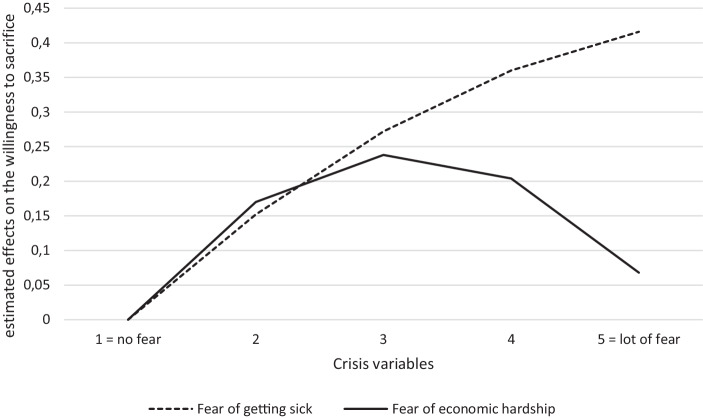
Effects of crisis variables on environmental willingness (estimated values, based on Model 4 of Table 3)^a^. (^a^The graphs show the estimated values of the willingness to sacrifice for the environment for the five possible answer categories (1 = no fear, …, 5 = a lot of fear) considering the B‑value and the squared term, i.e. 0.181 and −0.098 for the health dimension and 0.250 and −0.342 for the economic dimension)

The effect of economic worries is curvilinear (see Fig. 2). The least willing to make sacrifices for the environment are those respondents who are very worried about economic effects and those who are not worried at all. That those who fear economic hardship have a low willingness to sacrifice is in line with our hypothesis and comes as no surprise. Individuals who are struggling financially will not have the means to spend more money on the environment or to lower their living standards. The low willingness to sacrifice by those who have no economic worries needs further thought. Considering the values and socio-demographics of this particular group (additional analyses, available upon request from the authors) shows that they are often well-off and have hedonistic attitudes. We thus face the possible interpretation that one group is unconcerned about the societal impacts of the pandemic and does not care about the environment either. The health concern, on the other hand, had the expected result in the sense that lower worries are associated with a lower willingness to make sacrifices. Yet, this effect became insignificant when combined with environmental concern. Here, a possible interpretation is that we observe a general underlying notion of concern, which is in line with the benevolence and universalism dimension of Schwartz’s theory.

In sum, our analysis indicates some negative effects of the COVID-19 crisis on the willingness to sacrifice for the environment. Given the brevity of a research note, future research will have to elaborate on the implications of our findings on the underlying theoretical considerations. It will also be necessary to conduct additional studies, based on other data collection methods than online panels once the restrictions in fieldwork have waned. Finally, it will be interesting to see whether or not the pandemic has changed environmental behaviors and concerns in the long run or if rebound effects occur once the pandemic is over.

## Footnotes

1 We considered testing the three dimensions of willingness to sacrifice for the environment separately. The effects of the explanatory variables differ only in regard to income. It has a significant positive influence on the willingness to pay higher prices and taxes. The willingness to pay higher prices and taxes is higher for respondents with a higher net household income than for those with a lower income. In contrast, income has no influence on the willingness to cut back on living standards.

2 Due to the curvilinear relationship of the main variable (willingness) and the crisis variables, squared variables of each crisis variable are added. The results for a model that includes only the linear effects are reported below in Table 3.
